# The Effect of Training in the Preparatory and Competitive Periods on Trunk Rotational Power in Canoeists, Ice-Hockey Players, and Tennis Players

**DOI:** 10.3390/sports6040113

**Published:** 2018-10-09

**Authors:** Oliver Poór, Erika Zemková

**Affiliations:** 1 Department of Sports Kinanthropology, Faculty of Physical Education and Sport, Comenius University in Bratislava, 81469 Bratislava, Slovakia; oliverpoor55@gmail.com; 2 Sports Technology Institute, Faculty of Electrical Engineering and Information Technology, Slovak University of Technology in Bratislava, 81219 Bratislava, Slovakia; 3 Institute of Physiotherapy, Balneology and Medical Rehabilitation, University of Ss. Cyril and Methodius in Trnava, 91701 Trnava, Slovakia

**Keywords:** canoeists, ice-hockey players, tennis players, pre-season and in-season training, trunk rotations

## Abstract

This study evaluates changes in trunk rotational power at different weights and velocities after the preparatory and competitive periods in ice-hockey players, tennis players, and canoeists. The subjects performed trunk rotations to each side with a barbell of different weights placed on the shoulders (6, 10, 12, 16, 20, 22, and 26 kg) prior to and after 6 weeks of the preparatory period and 6 weeks of the competitive period. The results showed that mean power produced in the acceleration phase of trunk rotations increased significantly at weights from 10 to 26 kg or 6 to 26 kg after the preparatory and competitive periods in tennis players. The values obtained during trunk rotations with weights ≥12 kg also increased significantly after the preparatory period in ice-hockey players, whereas there were no significant changes after the competitive period. Similarly, the mean power during trunk rotations with weights ≥10 kg increased significantly only after the preparatory period in canoeists. Similar changes were observed for the peak power. These findings demonstrate that changes in trunk rotational power reflect the specificity of their training programs. This information may provide a basis for designing exercises focused on improvements of power produced during trunk rotations under loading conditions.

## 1. Introduction

The important role of the core for force generation and stabilization in most sports is being recognized. The “core” muscles can be visualized as a box with the abdominals in the front, paraspinals and gluteals in the back, the diaphragm as the roof, and the pelvic floor and hip girdle musculature as the bottom [1]. Core strength is related to the strength and power produced by these muscles, whereas the core stability is the capacity of the muscles to control the trunk position and its motion over the pelvis and leg to allow the force production to the terminal segment in integrated kinetic chain exercises [2].

Core strength and stability exercises have been used as performance-enhancing programs, preventative regimens, and to help with rehabilitation. However, there is conflicting and scarce scientific evidence on the effectivity of these exercises for the enhancement of athlete performance [3,4] or prevention and rehabilitation of injuries [5,6]. This is due to the lack of testing methods of core strength and stability.

The assessment of core stability requires incorporating coordination and balance. A good example is the lunge, during which the deep trunk muscles coordinate the spine, pelvis, and hips while thrusting the body forwards. Another example is the “clean and jerk” which requires strong muscles of the core and correct spinal alignment when lifting a heavy weight. Additionally, the ability of subjects to maintain the stable core while sitting or standing on unstable surfaces when lifting weight with the arms or legs is considered a test of core stability [7].

Field tests of core strength include the measurement of the amount of weight lifted, the number of repetitions performed, and the time required to maintain a neutral stable position [8]. In the laboratory, triaxial lumbar dynamometers are rare [9,10,11], therefore, isokinetic and isometric dynamometers are used [12,13]. However, the external validity of isokinetic strength and isometric endurance tests of trunk muscles for sport-specific tasks is ambiguous. While some authors have reported the relationship between athletic performance and measures of core strength [14,15], others have not [16,17,18]. Core strength does have a significant effect on an athlete’s ability to create and transfer forces to the extremities [19]. The association between the core muscles and limbs has been demonstrated in various lifting tasks, forehand and backhand strokes in tennis, overhead throwing in baseball, cycling and many other examples [20,21,22,23,24,25,26]. The effective execution of the tennis stroke or golf swing requires not only the rapid movement of the extremities but also the strength of trunk muscles to generate velocity and power during trunk rotations. Trunk muscles (erector spinae, abdominal oblique, and rectus abdominis) are particularly active during the acceleration phase of trunk rotations (e.g., the golf swing) with the trial-side abdominal oblique muscles showing the highest level of activity [27]. This emphasizes the importance of the core in the transfer of torques and momentum throughout the kinetic chain exercises. Deficiencies in this kinetic chain can affect athletic performance or increase the risk of injury [7]. Therefore, when assessing the core strength, it is crucial to take the demands of all muscles and joints in this kinetic chain into account.

Current tests used for assessing the effect of training focused on increasing core strength and stability are insufficient. These usually include the biomechanical analysis of technique, the cross-sectional training evidence, and experience of conditioning specialists. Additionally, the insufficient sensitivity and low reliability of tests evaluating the core strength limits their applications in practice. Another drawback is that these tests do not target the major spine stabilizers in spite of the fact that most of them are task specific.

There is a need to provide conditions for testing that are close to those used during sport-specific movement tasks. Usually, the resistance exercises with weights increasing stepwise up to the 1 repetition maximum (1 RM) are performed to obtain power-velocity and force-velocity curves or to analyse power and/or velocity to weight relationship. The velocity produced in the concentric phase of exercise decreases with increasing weights. However, the power increases from lower weights, and after reaching a peak, decreases toward higher weights. The optimal velocity that allows the production of the highest power depends on the ratio of slow and fast twitch muscle fibers [28] and, therefore, it could be hardly changed with training. However, the weight at which maximal values of power are achieved can increase with the training. Maximal values of power during bench presses and squats are achieved at 50–60% of 1 RM [29], whereas they are achieved during trunk rotations at 30–45% of 1 RM [30]. This variation in power produced at different loads in athletes of various specializations may be attributed to the specificity of their training programs. 

In order to verify this assumption, one should evaluate the effect of training programs on trunk rotational power in athletes whose performance requires the generation of strength at higher or lower velocities during trunk rotations (canoeists, tennis players, ice-hockey players, golfers, etc.) [31,32]. Therefore, this study evaluated the changes in the peak and mean power produced during trunk rotations with different weights after 6 weeks of the preparatory period and 6 weeks of the competitive period in ice-hockey players, tennis players, and canoeists. We hypothesized that the trunk rotational power increases at higher weights after the preparatory period, whereas it increases at lower weights after the competitive period due to the differential training loads used. 

## 2. Materials and Methods

### 2.1. Participants

Groups of male canoeists, ice-hockey players, and tennis players volunteered to participate in the study (Table 1). All of them were active in a particular sport at a competitive level. The athletes also had experience with resistance training including exercises strengthening core muscles. The procedures followed were in accordance with the ethical standards on human experimentation stated in compliance with the 1964 Helsinki Declaration and its later amendments.

### 2.2. Training and Testing

Subjects underwent 6-week training during the preparatory period followed by 6-week training in the competitive period. According to Baechle and Earle [33], the off-season is the period between the postseason and 6 weeks prior to the contest of the next year’s season. This season includes most of the preparatory period and can be divided into multiple shorter mesocycles. The preseason period occurs next, leads up to the first contest, and commonly contains the late stages of the preparatory period and the first transition period. The competition, in-season period contains all the contests scheduled for that year, including any tournament games. 

Conditioning training in the preparatory period was focused on the improvement of movement speed and muscle strength and power. The training consisted of three to four 45–60 min sessions or two 90 min sessions. In the competitive period, conditioning training was carried out 2–3 times per week, whereas sport-specific training was carried out 3–5 times per week depending on the competition schedule. Examples of the training program in both periods are included in Table 2 and Table 3. Exercises during the preparatory period were performed with heavier weights in order to gain greater muscle strength. This is an example of the strength training:

- warm up: 5 min of rope skipping,
- resistance exercises (repetitions × sets × rest): back squat (6 × 3 × 90 s), bench press (8 × 3 × 90 s), parallel bars push-up with an additional load (6 × 3 × 90 s), sit-ups with 5 kg medicine ball (20 × 3 × 60 s), seated lat pulldown (8 × 3 × 90 s), seated row (8 × 3 × 90 s), seated shoulder dumbbells press (8 × 3 × 90 s),
- cooldown: 5 min of stretching.

Training in the competitive period mainly included sport-specific exercises performed with maximal intensity. This is an example of the strength training:

- warm up: 5 min of cycling,
- resistance exercises (repetitions × sets × rest): one leg squat (10 × 3 × 90 s), push up (15 × 3 × 90 s), lunge (20 × 3 × 60 s), chin to bar (8 × 3 × 90 s), squat to jump (10 × 3 × 90 s),
- cooldown: 5 min of stretching.

Sport-specific exercises included tennis shots with therabands and medicine ball throws imitating forehand or backhand shots, movements imitating hockey shots using “TRX rib training” or a pulley machine, and rotational movements imitating paddle shots using a pulley machine.

**Figure 1:**
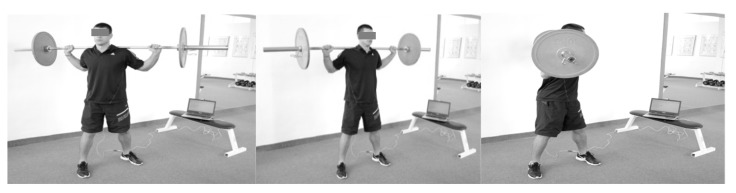
Standing rotations of the trunk with a barbell of different weights.

Participants were tested prior to and after the preparatory and competitive periods. The experimental protocol was provided according to the one described in the previous study [34], however, by using different weights (6, 10, 12, 16, 20, 22 and 26 kg) (Figure 1). Please see the supplementary file. 

**Figure 2:**
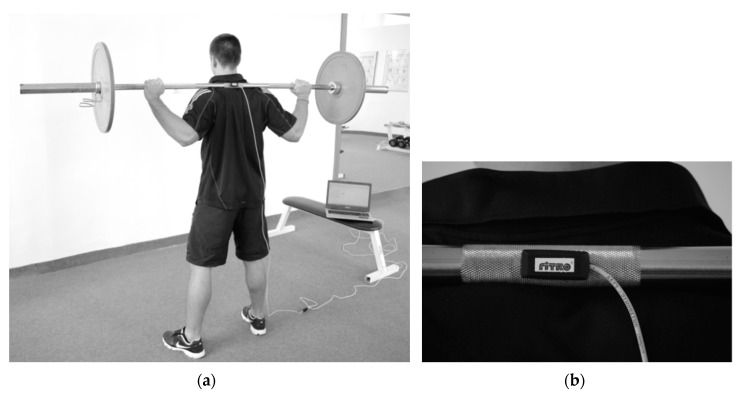
The assessment of trunk rotational velocity and power (**a**) using the FiTRO Torso Premium (**b**).

Basic biomechanical parameters during trunk rotations were monitored using the FiTRO Torso Premium (FiTRONiC, Bratislava, Slovakia), Figure 2, as previously described [34]. Please see the supplementary file. This diagnostic system allows for the measuring and analyzing of parameters in the acceleration and the deceleration phase, as well as in whole rotational phase. In the present study, the peak power and mean power produced in the acceleration phase of trunk rotations at different weights and velocities were analysed. 

### 2.3. Statistical Analyses

Data were analysed using the statistical program SPSS for Windows, version 22.0 (SPSS, Inc., Chicago, IL, USA). A Wilcoxon signed-rank test was employed to determine the significance of changes in the trunk rotational power prior to (pre-preparation) and after 6 weeks of the preparatory period (post-preparation) and 6 weeks of the competitive period (post-competition) in hockey players, tennis players, and canoeists. The Mann–Whitney U test was used to determine the significance of changes in the trunk rotational power between the preparatory and the competitive period in these groups of athletes. The significance level was set at *p* < 0.05. All data are presented as means of particular groups of athletes and standard deviations. Pre-post training changes in trunk rotational power are expressed in percentages. 

## 3. Results

**Figure 3:**
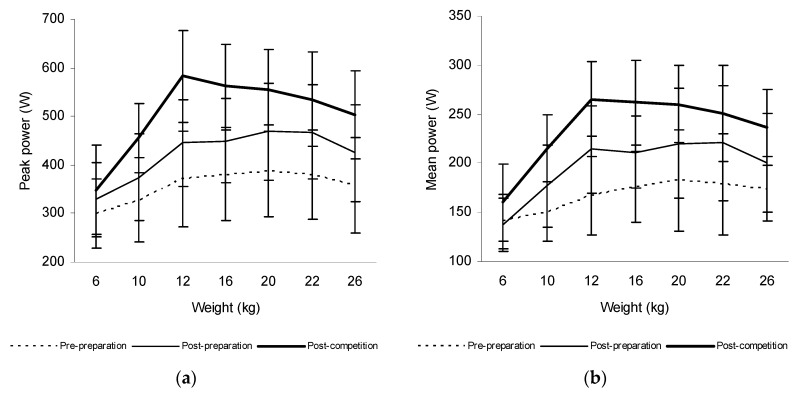
The peak power (**a**) and mean power produced during trunk rotations (**b**) at weights from 6 to 26 kg prior to and after 6 weeks of the preparatory period and 6 weeks of the competitive period in tennis players.

The mean power in the acceleration phase of the trunk rotations increased significantly at weights of 10 kg (14.6%, *p* = 0.012), 12 kg (22.0%, *p* < 0.001), 16 kg (16.6%, *p* < 0.001), 20 kg (17.8%, *p* = 0.006), 22 kg (19.0%, *p* = 0.008), and 26 kg (13.1%, *p* = 0.014) and the peak power at weights of 12 kg (17.0%, *p* = 0.043), 16 kg (15.9%, *p* = 0.028), and 26 kg (15.9%, *p* = 0.043) after the preparatory period in tennis players. There was also a significant increase of the mean power produced during trunk rotations at weights of 6 kg (14.2%, *p* = 0.012), 10 kg (17.8%, *p* < 0.001), 12 kg (19.2%, *p* < 0.001), 16 kg (19.3%, *p* < 0.001), 20 kg (14.6%, *p* = 0.038), 22 kg (12.2%, *p* = 0.050), and 26 kg (15.5%, *p* = 0.004) and the peak power at 6 kg (12.5%, *p* = 0.018), 10 kg (17.8%, *p* = 0.018), 12 kg (23.5%, *p* = 0.018), 16 kg (20.1%, *p* = 0.018), 20 kg (13.5%, *p* = 0.018), 22 kg (12.5%, *p* = 0.018), and 26 kg (15.6%, *p* = 0.018) after the competitive period in these athletes (Figure 3).

**Figure 4:**
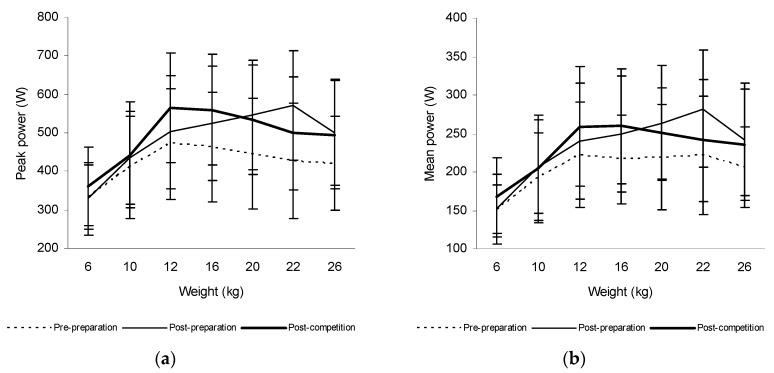
The peak power (**a**) and mean power produced during trunk rotations (**b**) at weights from 6 to 26 kg prior to and after 6 weeks of the preparatory period and 6 weeks of the competitive period in ice-hockey players.

Furthermore, a significant increase of the mean power produced during trunk rotations at weights of 12 kg (7.6%, *p* = 0.019), 16 kg (13.0%, *p* < 0.001), 20 kg (13.3%, *p* = 0.003), 22 kg (21.4%, *p* < 0.001), and 26 kg (14.7%, *p* < 0.001) and the peak power at 20 kg (13.7%, *p* = 0.020), 22 kg (25.3%, *p* = 0.047), and 26 kg (16.0%, *p* = 0.020) was also observed after the preparatory period in ice-hockey players. However, their values did not change significantly after the competitive period in these athletes (Figure 4). 

**Figure 5:**
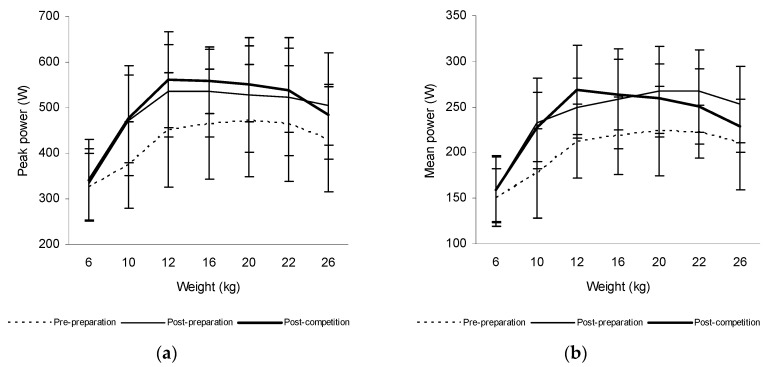
The peak power (**a**) and mean power produced during trunk rotations (**b**) at weights from 6 to 26 kg prior to and after 6 weeks of the preparatory period and 6 weeks of the competitive period in canoeists.

Similarly, the mean power produced during trunk rotations increased significantly at weights of 10 kg (23.5%, *p* < 0.001), 12 kg (11.5%, *p* = 0.004), 16 kg (15.6%, *p* < 0.001), 20 kg (19.5%, *p* < 0.001), 22 kg (16.7%, *p* = 0.002), and 26 kg (16.6%, *p* = 0.001) and the peak power at 10 kg (20.6%, *p* = 0.012), and 12 kg (15.8%, *p* = 0.018) after the preparatory period only in the group of canoeists (Figure 5).

**Figure 6:**
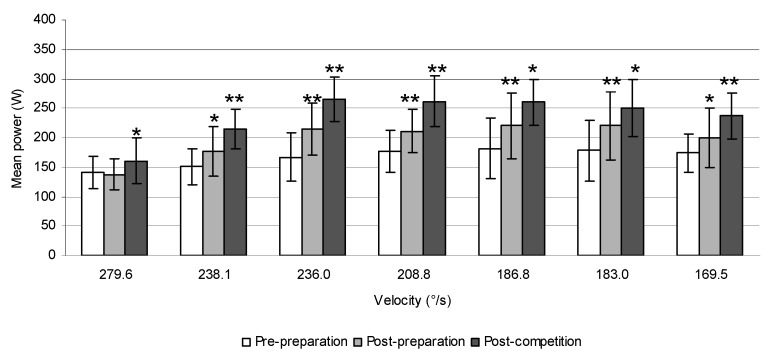
The mean power produced during trunk rotations at different velocities prior to and after 6 weeks of the preparatory period and 6 weeks of the competitive period in tennis players (* *p* ≤ 0.05, ** *p* ≤ 0.01).

**Figure 7:**
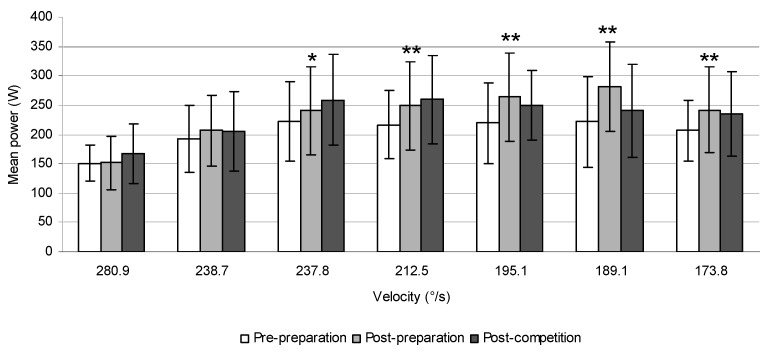
The mean power produced during trunk rotations at different velocities prior to and after 6 weeks of the preparatory period and 6 weeks of the competitive period in ice-hockey players (* *p* ≤ 0.05, ** *p* ≤ 0.01).

**Figure 8:**
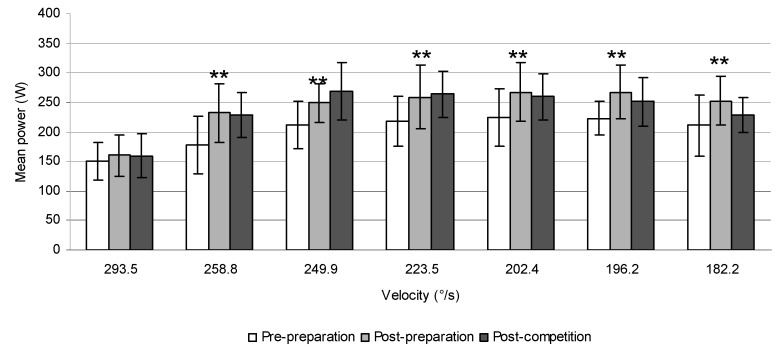
The mean power produced during trunk rotations at different velocities prior to and after 6 weeks of the preparatory period and 6 weeks of the competitive period in canoeists (* *p* ≤ 0.05, ** *p* ≤ 0.01).

The mean values of power at different velocities in tennis players, ice-hockey players, and canoeists are displayed in Figure 6, Figure 7 and Figure 8.

## 4. Discussion

The mean power produced during trunk rotations increased significantly at weights of 10, 12, 16, 20, 22, and 26 kg and the peak power at weights of 12, 16, and 26 kg after the preparatory period in tennis players. There was also a significant increase of its values at weights of 6, 10, 12, 16, 20, 22, and 26 kg and the peak power at 6, 10, 12, 16, 20, 22, and 26 kg after the competitive period in these athletes. The mean power increased significantly also at weights of 12, 16, 20, 22, and 26 kg and the peak power at 20, 22, and 26 kg after the preparatory period in ice-hockey players, whereas its values did not significantly change after the competitive period. Likewise, the mean power increased significantly at weights of 10, 12, 16, 20, 22, and 26 kg and the peak power at 10 and 12 kg after the preparatory period only in a group of canoeists. These finding supported our hypothesis that trunk rotational power increases mainly at higher weights after the preparatory period in all three groups of athletes. However, its values also increased significantly at all weights used after the competitive period in tennis players, whereas no significant changes were observed in ice-hockey players, and canoeists. 

These improvements in the trunk rotational power after the preparatory, as well as the competitive period in tennis players and after only the preparatory period in ice-hockey players, and canoeists, may be attributed to the specificity of the training applied. The training in the preparatory period in tennis players was mainly focused on the improvement of muscle strength using resistance exercises, such as squats, bench presses, and deadlifts, as well as trunk rotation exercises with barbells and medicine balls. The training in the competitive period in tennis players included game-specific exercises and competitive matches, in addition to balance and core stability exercises in order to avoid the risk of injury. Additionally, ice-hockey players used higher weights in their strength training in the preparatory period with the aim of increasing muscle strength and power. On the other hand, they used lower weights in their competitive period and performed exercises with maximal effort in the concentric phase. This training period also incorporated conditioning and game-specific skills on the ice in addition to hockey matches. Similarly, the workouts in the preparatory period in canoeists included resistance exercises with higher weights in comparison with lower weights used in the competitive period. However, in both cases, attention was paid on increasing the speed of movement. The competitive period mainly incorporated training of sport-specific skills and canoeing techniques in the water.

These findings indicate that such an assessment is sensitive in revealing training-induced changes in the trunk rotational power. Previous studies also showed that mean values of velocity and power produced during trunk rotation are able to reveal within- and between-group differences [35,36]. More specifically, the mean power produced with a weight of 20 kg was significantly higher in tennis players than golfers, in rock and roll dancers than in ballroom dancers, and in judoists than in wrestlers. The mean velocity was also significantly higher in tennis players than in golfers; however, this applied only when the weight of 1 kg was used. A significantly higher trunk rotational velocity with both 1 and 20 kg was also found in rock and roll dancers compared to ballroom dancers. On the other hand, these values did not differ significantly between judoists and wrestlers with weights of 1 and 20 kg. The comparison of the trunk’s rotational power with 20 kg and its velocity with 1 and 20 kg between individuals showed greater values in the ice-hockey player than in the karate competitor, in the canoeist than in the rower, and in the weightlifter than in the bodybuilder [35,36]. These group and individual differences in the trunk rotational velocity and power can be ascribed to the specificity of their training programs involving movements of the trunk at different velocities under various loading conditions.

Hence, the assessment of trunk rotational power should be considered an integral part of functional diagnostics in sports that require athletes to produce a high force in a short time. It may provide important information on the efficiency of the training program, as well as differences in the trunk rotational power among athletes of various sports.

Usually, the effect of core stability or core strength training on athletic performance is evaluated. Although many studies documented that a strong and stable core is a foundation for athletic performance, the systematic review by Reed et al. [4] showed mixed results. According to the authors, there are several challenges to the assessment of the effects of core training. The core training is usually a part of a larger training regimen and, therefore, it is difficult to isolate the effect of core exercises on performance. Furthermore, the effects associated with recreationally active subjects cannot be translated to highly trained competitive athletes. It is also difficult to perform a randomized controlled trial when working with a group of athletes in team sports. Our study is unique in evaluating the changes in trunk rotational power at lower and higher weights (from 6 to 26 kg) after two training periods (preparatory and competitive) in three groups of competitive athletes (tennis players, ice-hockey players, and canoeists).

The limitation of this study is that the trunk rotational power was not measured in a sitting position in canoeists, which would provide more specific testing conditions for these athletes. On the other hand, it would not allow us to compare the power produced during trunk rotations with tennis and ice-hockey players. Although seated resistance exercises are more safe and stable, they are less efficient in power production than those performed in a standing position. An example is the rotational exercises of the trunk with an additional load. The trunk rotational power was found to be significantly higher during standing than in seated rotations of the trunk with weights ≥10.5 kg [34]. This can be attributed to a higher range of trunk rotational motion during standing than whilst sitting, which allows for the acceleration of the movement at the beginning of the rotation more forcefully. This resulted in a higher velocity of trunk rotations and also of power outputs. The legs can help to perform trunk rotational movements more effectively, namely, when the weight is heavy, although maintaining balance may be more difficult while standing compared to sitting. 

Trunk rotations performed in a seated position reduce the involvement of the legs and the contribution of thoracic/hip mobility to the rotational velocity of the upper body. The reduced range of motion of the thoracic spine and the hips, which allows the greatest rotation because of the orientation of the joints [37], can contribute to the lower velocity of the trunk movement and subsequently affect ball or puck velocity in tennis and hockey. These sports require the production of explosive movement in the oblique plane [38]. The force is transferred from the proximal to the distal segments. Because of the kinetic linkage between these segments [39], the trunk rotational mobility may play an essential role in the production of velocity and power. This velocity and power transference of the hips and upper trunk may be important to the velocity of the hockey stick and tennis racquet. Hence, the test that most closely replicates movements of the upper/lower body, i.e., standing trunk rotations for ice-hockey and tennis players and seated trunk rotations for canoeists, should be used for testing their sport-specific power performance and its changes during the training. The present study demonstrated that the test used is able to sensitively reveal the effects of different training periods on trunk rotational power and can be implemented in practice.

## 5. Conclusions

The study showed that the mean power produced during trunk rotations increased significantly at weights from 10 to 26 kg and the peak power at 12, 16, and 26 kg after the preparatory period in tennis players, whereas at weights from 6 to 26 kg after the competitive period. Their values increased significantly also during trunk rotations with weights ≥12 kg and ≥20 kg respectively after the preparatory period in ice-hockey players. However, no significant changes in their values after the competitive period in these athletes were found. Similarly, the mean and peak values of power significantly increased during trunk rotations with weights ≥10 kg and 10 and 12 kg, respectively, after only the preparatory period in canoeists. These findings demonstrate that changes in the trunk rotational power reflect the specificity of their training programs, to some extent. A better understanding of the role that sport-specific exercises plays in the enhancement of power produced during trunk rotations would enable us to design more functional training programs. 

## Figures and Tables

**Table 1:** The characteristics of the groups of athletes (mean ± SD).

Groups of Athletes	N	Years of Competition	Level of Competition	Years of Experience with Resistance Training	Age (years)	Height (cm)	Body Mass (kg)
Canoeists	8	7.7 ± 3.4	International, World championships	3.7 ± 1.8	21.0 ± 1.9	181.1 ± 5.5	78.8 ± 13.6
Ice-hockey players	15	14.6 ± 1.4	National league	6.9 ± 2.0	19.9 ± 1.1	182.7 ± 4.6	81.2 ± 5.7
Tennis players	7	6.0 ± 2.4	Amateur, Regional tournaments	5.4 ± 3.0	25.9 ± 1.1	178.6 ± 4.1	76.0 ± 6.4

**Table 2:** An example of the pre-season conditioning training in tennis players, ice-hockey players, and canoeists.

**Tennis Players**	**Monday**	**Tuesday**	**Wednesday**	**Thursday**	**Friday**	**Saturday**	**Sunday**
Morning or afternoon	Strength training	Strength training	Tennis game	Strength training	Fitness training	Tennis game	Tennis game
General/specific training (minutes)	60/0	60/0	0/60	60/0	30/0	-	-
**Ice-Hockey Players**	**Monday**	**Tuesday**	**Wednesday**	**Thursday**	**Friday**	**Saturday**	**Sunday**
Morning (M)	Strength training	Strength training	-	Strength training	-	Individual physical activities	No training
Afternoon (A)	Fitness games; Endurance and agility training	Fitness games; Strength and agility training	Swimming	Fitness games; Agility training	Outdoor cycling	-	No training
General/specific training (minutes)	75(M) + 120(A)/0	75(M) + 120(A)/0	120(A)/0	75(M) + 120(A)/0	120(A)/0	-	-
**Canoeists**	**Monday**	**Tuesday**	**Wednesday**	**Thursday**	**Friday**	**Saturday**	**Sunday**
Morning or afternoon	Strength training	Strength training	No training	Strength training	Active recovery phase	No training	Active recovery phase
General/specific training (minutes)	90/0	90/0	-	90/0	60/0	-	90/0

**Table 3:** An example of in-season conditioning training in tennis players, ice-hockey players, and canoeists.

**Tennis Players**	**Monday**	**Tuesday**	**Wednesday**	**Thursday**	**Friday**	**Saturday**	**Sunday**
Morning or afternoon	Active recovery phase	Tennis game	Active recovery phase	Tennis game	Active recovery phase	Tennis match	Tennis match
General/specific training (minutes)	60/0	0/60	60/0	0/60	60/0	-	-
**Ice-Hockey Players**	**Monday**	**Tuesday**	**Wednesday**	**Thursday**	**Friday**	**Saturday**	**Sunday**
Morning (M)	-	Fitness training; Ice-hockey training	-	-	-	-	-
Afternoon (A)	Ice-hockey training; Strength training	Active recovery phase	Ice-hockey training; Strength training	Ice-hockey training; Upstairs running	Ice-hockey training; Strength training	Hockey match	Hockey match
General/specific training (minutes)	30(A)/75	60(M)/15	30(A)/60	30(A)/75	30(A)/45	-	-
**Canoeists**	**Monday**	**Tuesday**	**Wednesday**	**Thursday**	**Friday**	**Saturday**	**Sunday**
Morning or afternoon	Canoeing training	Strength training	Canoeing training	Strength training	Canoeing training	Canoeing training	No training
General/specific training (minutes)	0/120	60/0	0/120	60/0	0/120	0/120	-

## Supplementary Materials

The following are available online at http://www.mdpi.com/2075-4663/6/4/113/s1.
