# Supplementary material for: The Effect of Training in the Preparatory and Competitive Periods on Trunk Rotational Power in Canoeists, Ice-Hockey Players, and Tennis Players
Source: Sports (Basel). 2018 Oct 9;6(4):113. doi: 10.3390/sports6040113 (PMC6316482; doi:10.3390/sports6040113)
Supplement: Supplementary file 1 [file sports-06-00113-s001.pdf]

sports

Supplementary file

The effect of sport-specific training in the preparatory and competitive periods on trunk rotational power

Experimental protocol

Following the warm-up, participants were exposed to a familiarization trial during which they performed standing trunk rotations in a slow and controlled manner while keeping the back straight. Afterwards, they completed two repetitions of trunk rotations to each side in a standing position with a bar weight of 6, 10, 12, 16, 20, 22 and 26 kg placed on their shoulders (Figure 1). They were instructed to perform trunk rotations with maximal effort in the acceleration phase. Emphasis was placed on the proper position of the body during the exercise. They stood with their feet wider than shoulder width apart and toes slightly pointed outwards while holding the barbell on the shoulders. They rotated their torso forcefully from the right (or the left) towards the opposite side until the body reached the end position, and then they slowly returned to the starting position. The test was then repeated for the opposite side of the body. They had to engage their core muscles to stiffen the torso and stabilize the spine. A laboratory assistant made sure that participants remained upright throughout the movement and that their head, chest and torso were aligned over their hips.

Assessment of trunk rotational power and velocity

The FiTRO Torso Premium (FiTRONiC, Slovakia) consists of an inertia measurement unit in a small box with an integrated USB interface and software (Figure 2). While inserted on the barbell axis, the sensor unit registers instant angle of rotation movement. Calculations of force and power are based on Newton´s second law of mechanics. Force produced to accelerate and decelerate a rotation movement is obtained as a product of barbell mass and acceleration of its center of gravity (CoG). Angular acceleration is obtained by derivation of angular velocity. For the transformation of angular velocity and acceleration into their real values, a rotation radius (distance between rotation axis and barbell mass CoG) is used. Power is calculated as a product of force and velocity.
